# Comparing clinicopathological features and prognosis of primary pulmonary invasive mucinous adenocarcinoma based on computed tomography findings

**DOI:** 10.1186/s40644-019-0236-2

**Published:** 2019-07-10

**Authors:** Kai Nie, Wei Nie, Yu-Xuan Zhang, Hong Yu

**Affiliations:** 1 Department of Imaging and Nuclear Medicine, Changzheng Hospital, Second Military Medical University, Shanghai, 200003 China; 2 Department of Radiology, Shanghai Chest Hospital, Shanghai Jiaotong University, No.241 Huaihai West Road, Xuhui Area, Shanghai, 200030 China; 3 Department of Respiration, Shanghai Chest Hospital, Shanghai Jiaotong University, Shanghai, 200030 China; 4 School of pharmacy, Queen’s University Belfast, Medical Biology Centre, 97 Lisburn Road, Belfast, BT9 7BL Northern Ireland, UK

**Keywords:** Lung cancer, Invasive mucinous adenocarcinoma, Computed tomography, Disease-free survival, Prognosis

## Abstract

**Objective:**

To evaluate the relationship between clinicopathologic characteristics and prognosis in patients with invasive mucinous adenocarcinoma (IMA) of the lung.

**Methods:**

A total of 68 patients who underwent surgical resection for primary lung IMA were reviewed during the period of 2009 and 2017. Tumors were classified as solitary-type or pneumonic-type according to the computed tomography (CT) findings. Cox proportional hazards model was used to assess the effects of clinicopathological characteristics on univariate and multivariable analyses of disease-free survival (DFS).

**Results:**

Solitary-type was found in 54 patients, while pneumonic-type was found in 14 patients. The patients’ age varied between 56 and 68 years (patients’ median age was 61 years). Besides, 50 patients had T1/T2 tumor stage (73.5%). Compared with solitary-type, higher T stage, N stage, and pathological stage (*P* < 0.001) were found in pneumonic-type. Moreover, the survival analysis showed that the pneumonic-type had a significantly poorer DFS compared with solitary-type (*P* = 0.004). Univariate analysis showed that pneumonic pattern on CT scan, T stage, pathologic stage, and thyroid transcription factor-1 (TTF-1) were significant predictive factors of survival (*P* = 0.011, 0.014, 0.013, 0.029, respectively). Multivariate analysis further indicated that pneumonic-type was the only independent prognostic factor for poor survival [hazard ratio (HR) = 6.764, 95% confidence interval (CI): 1.563–29.269, P = 0.011].

**Conclusions:**

Based on CT findings, the solitary-type IMA is associated with a lower stage and better prognosis compared with the pneumonic-type IMA.

## Background

Lung cancer is a major cause of cancer-related deaths worldwide. Non-small cell lung cancer (NSCLC) accounts for more than 80% of all lung cancers, among which adenocarcinoma is the most common pathological subtype. Invasive mucinous adenocarcinoma (IMA), formerly known as mucinous bronchioloalveolar carcinoma (BAC), is relatively rare adenocarcinoma, accounting for only 2–5% of lung invasive adenocarcinomas [1]. According to the International Association for the Study of Lung Cancer (IASLC)/American Thoracic Society (ATS)/European Respiratory Society (ERS) classification system for lung adenocarcinoma published in 2011, due to unique pathological, radiological, and prognostic characteristics, this type of disease has been separated from the adenocarcinomas formerly classified as nonmucinous BAC [2]. In 2015, the World Health Organization (WHO) also classified IMA as an invasive adenocarcinoma subtype [3].

As a separate category of lung adenocarcinoma, IMA is diagnosed based on tall columnar cell morphology with abundant intracellular or extracellular mucus and with invasive adenocarcinoma patterns, such as lepidic, acinar, papillary, micropapillary, and solid predominant patterns. Based on the percentage of mucinous pattern, IMA can be divided into two groups: pure mucinous (> 90% invasive mucinous pattern, lepidic predominant pattern) and mixed mucinous/nonmucinous (≧ 10% of nonmucinous invasive component) [1]. Several studies have demonstrated that the new lung adenocarcinoma classification has a clear prognostic significance [4–6], suggesting that IMAs are typically associated with poor survival outcomes compared with non-mucinous lepidic, acinar, and papillary predominant subtypes of lung adenocarcinoma [7, 8]. Lymph node involvement and distant metastasis are less frequent in IMA compared with other subtypes of adenocarcinoma. Nevertheless, the aerogenous spread and satellite tumors surrounding the main lesion frequently occur in patients with IMA [9]. In addition, a number of studies [10–12] have discussed different groups of IMA based on computed tomography (CT), in which their results indicated that these groups were closely associated with the clinical outcomes. However, due to low incidence of this subtype, the number of patients included in those studies was relatively small, and the patients with pathological diagnosis of IMA were not differentiated from pure mucinous or mixed mucinous/nonmucinous IMA. Therefore, the present study investigated the association between clinicopathological features and prognosis of subtypes of lung primary IMA based on CT findings.

## Patients and methods

This retrospective study was approved by the Institutional Review Board of Shanghai Changzheng Hospital (Shanghai, China), and the requirement for informed consent was waived.

### Patients’ selection

A total of 730 patients who underwent surgical resection for lung adenocarcinoma at our hospital between October 2009 and August 2017 were recruited in this study. Inclusion criteria were: 1) patients with mucinous adenocarcinoma confirmed on surgical resection, in which baseline CT examination of chest was performed before surgical resection in our institution; 2) clinical and imaging data available in the electronic medical records. These patients did not undergo radiotherapy or chemotherapy prior surgery. Exclusion criteria were as follows: 1) patients with multiple primary lung cancer; 2) patients with history of other malignancies; 3) patients who diagnosed with new subtypes of mucinous adenocarcinoma in situ (m-AIS) or mucinous minimally invasive adenocarcinoma (m-MIA); 4) patients with mixed invasive mucinous/nonmucinous adenocarcinoma (those patients had ≧ 10% of nonmucinous components and had worse survival outcome compared with those with pure IMA or non-mucinous adenocarcinomas) [13, 14]. For patients who diagnosed with BAC or mucinous (colloid) carcinoma according to the 2004 lung adenocarcinoma classification system released by the WHO, all the slides of the resected specimens were re-inspected by two experienced lung pathologists.

All included tumors were pathologically confirmed as pure invasive mucinous adenocarcinoma according to the 2015 lung adenocarcinoma classification system released by the WHO. Three additional patients with clinical stage IV IMA were excluded because as were treated with chemotherapy rather than curative surgery due to the multiple extrathoracic metastases. Ultimately, 68 patients with surgically resected primary lung pure IMA were included in the present study.

### Evaluation of CT findings

**Fig. 1:**
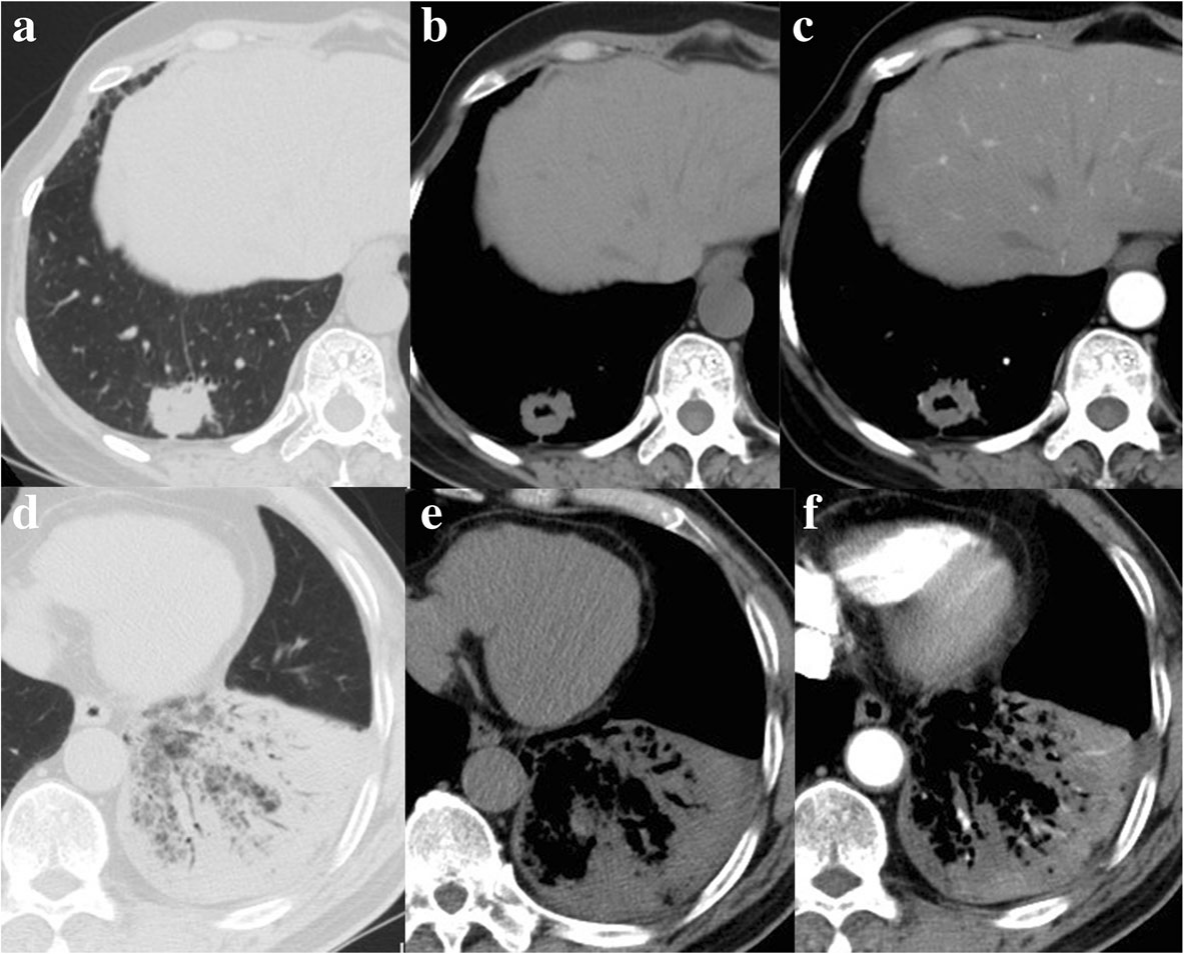
**a-c** A53-year-old female who presented solitary-type IMA on plain and contrast-enhanced computed tomography scans. **d-f** A73-year-old male who presented pneumonic-type IMA on plain and contrast-enhanced computed tomography scans

**Fig. 2:**
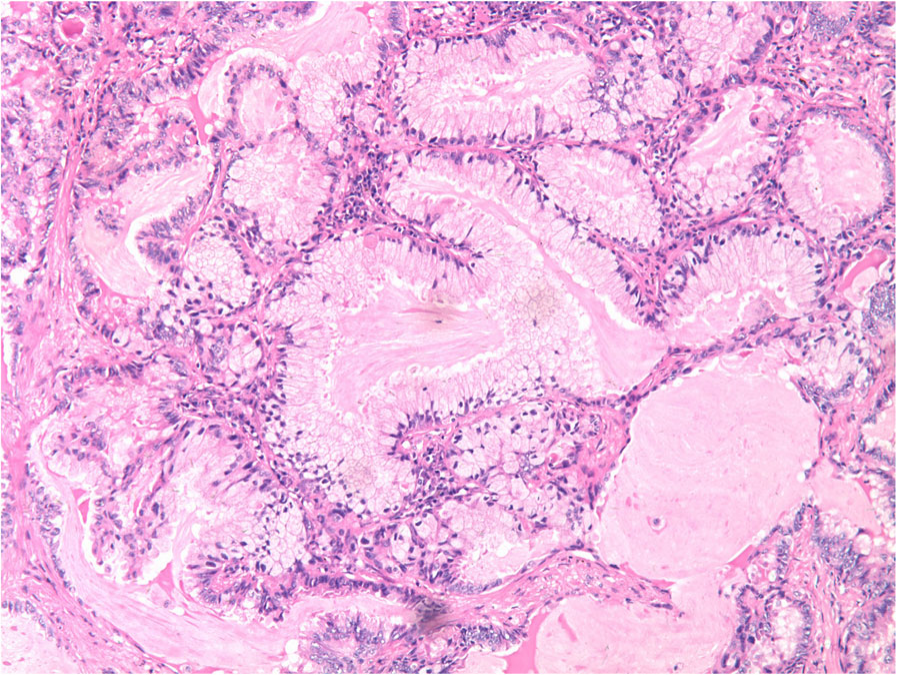
Histologic specimen showing tall columnar cells with basalnuclei and rich intracellular mucus (hematoxylin and eosin stain; original magnification ˣ 100)

All data were obtained using multi-detector CT scanner (Aquilion 16, Toshiba, Japan or Brilliance 256, Philips Healthcare, The Netherlands) in the craniocaudal direction during inspiration by using the following scanning parameters: X-ray tube voltage, 120 kVp; automatic tube currents; rotation speed, 0.5 s. CT slice thickness was as follows: 0.625 mm (6 patients, 8.9%), 1 mm (28 patients, 41.1%), and 5 mm (34 patients, 50%). Ioversol was used as a contrast medium at a flow rate of 3 ml/s through the elbow median vein at a dose of 1.5 ml/kg. Vascular phase and parenchymal phase images were obtained at 20~25 and 75~90 s, respectively. Two radiologists (with more than 15 years of experience on imaging of thoracic disease) independently assessed the CT findings based on tumor morphology on the mediastinal (window level,40 Hounsfield units; width, 400 Hounsfield units) and pulmonary window level settings (window level, − 600 Hounsfield units; width, 1,600 Hounsfield units). Consensus was achieved to make a final decision when disagreement in classification occurred between radiologists. Based on preoperative CT data, 68 IMAs were classified into two groups: 1): solitary-type, which was defined as solitary nodule or mass (Fig. 1 a-c); 2) pneumonic-type, that was defined as consolidation without definable shape and distributed along the lung lobe or lung segment, and sometimes with air bronchogram (Fig. 1 d-f) [11]. Other CT characteristics, such as site and number of involved lobes and tumor size were retrospectively assessed as well.

### Pathologic analysis

Pathologic diagnoses were carried out based on the 2011 IASLC/ATS/ERS classification system for lung adenocarcinoma. According to the 2015 WHO criteria, tumors with ≧ 90% mucinous cells were defined as pure IMA. The surgically resected specimens were formalin-fixed, paraffin-embedded, and stained with hematoxylin and eosin (H&E). All slides of the surgically resected specimens were interpreted by two experienced lung pathologists based on the consensus at the diagnosis of IMA. All slides representing the maximal surface area of the tumor in each patient were reviewed. The tall columnar cells with abundant intracellular mucus represent IMA (Fig. 2). The stages of all IMAs were evaluated based on the 8th edition of the tumor-node-metastasis (TNM) staging classification published by the Union for International Cancer Control (UICC) and the American Joint Committee on Cancer (AJCC) [15]. Markers of pulmonary differentiation were observed on one slice of tumor tissue per case using immunohistochemistry analysis. The main makers included nuclear staining for thyroid transcription factor-1 (TTF-1), cytoplasmic staining for cytokeratin 7 (CK7), and cytokeratin 20 (CK20), and positive expression was identified as staining of the cytoplasm or nucleus, which was detected in 10 fields of vision under high magnification (× 400). The interpretation of the results of immunohistochemistry was based on a consensus review by two pathologists.

### Follow-up

The patients were scheduled for follow-up every 1 to 3 months in the first year after tumor resection and at 6 months of intervals thereafter. Thoracic and abdominal multi-detector CT scans were performed at least once a year after surgery. The contrast-enhanced CT examination or biopsy was undertaken when recurrence was suspected during follow-up. If patients were not followed-up at our hospital, we recorded their survival status by telephone as well as the results of their previous examinations carried out in other hospitals.

### Statistical analysis

Categorical data were examined using the χ^2^ test and Fisher’s exact test. Non-normally distributed continuous variables were examined using Mann-Whitney U test. Disease-free survival (DFS) was calculated as the interval between surgery and first disease recurrence, including local-regional recurrence and distant metastasis, or death from any causes. The survival time was compared using the Kaplan-Meier method and log-rank test. Cox proportional hazards model was used to assess the effects of clinical and pathological characteristics on univariate and multivariate analyses of DFS. Multivariate analysis was performed using backward Cox proportional hazards regression with a step-down method. Variables with *P*-values < 0.10 on univariate analysis were used as input variables for multivariate analysis. The statistical analysis was performed using SPSS 22.0 software (IBM, Armonk, NY, USA), and *P* < 0.05 was considered statistically significant.

## Results

### Association between patients’ clinicopathological characteristics and CT findings

Table 1 shows the patients’ clinical features according to the type of tumor based on CT findings. Among the 68 IMA patients, 41 (60.3%) patients were female, and 27 (39.7%) patients were male. Solitary-type tumor was diagnosed in 54 patients, and 14 patients had pneumonic-type adenocarcinoma. The median age of the patients with solitary-type was 61 years (range, 56–68 years), and that of patients with pneumonic-type adenocarcinoma was 63 years (range, 53.5–70.5 years). There was no significant difference in demographic data. In addition, the majority of IMA patients had no obvious symptoms. Coughing out white sputum was the main symptom found in 18 patients (33.3%) with solitary-type, and six patients (42.9%) with pneumonic-type, however, no significant difference was found (*P* = 0.726). Besides, 50 (73.5%) tumors were in T1/T2 stage, and 60 (88.2%) were in N0 stage. Based on the CT findings, T and N stages significantly differed between the two types of patients with IMA. The pneumonic-type was associated with a higher pathological stage (*P* < 0.001) and relatively high T stage and N stage (P < 0.001, *P* = 0.008, respectively). Moreover, in our series, high expression of TTF-1 was found in 32/54 (59.3%) cases with solitary-type, while only one patient with pneumonic-type showed positive TTF-1 expression (*P* = 0.001). Additionally, positive CK7 expression was identified in 66 cases (Table 2).

**Table 1:** Clinical characteristics of tumor type based on computed tomography findings

Characteristics	Total No. (%)	Solitary type No.	Pneumonic type No.	*P* value
Total of patients	68	54	14	
Age				0.885
Median (interquartile range)	61 (12)	61 (12)	63 (17)	
Gender				0.339
Male	27 (39.7)	23	4	
Female	41 (60.3)	31	10	
Smoking history				1.000
Positive	20 (29.4)	16	4	
Negative	48 (70.6)	38	10	
Symptom				0.726
Positive	24 (35.3)	18	6	
Negative	44 (64.7)	36	8	
Type of surgery				0.229
Wedge resection	7 (10.3)	7	0	
Lobectomy	59 (86.8)	46	13	
Bilobectomy	2 (2.9)	1	1

**Table 2:** Pathologic characteristics of tumor type based on computed tomography findings

Characteristics	Total No. (%)	Solitary type No.	Pneumonic type No.	*P* value
Total of patients	68	54	14	
Pleural visceral invasion				0.884
Yes	16 (23.5)	12	4	
No	52 (76.5)	42	10	
T stage				< 0.001
T1/T2	50 (73.5)	48	2	
T3/T4	18 (26.5)	6	12	
N stage				0.008
N0	60 (88.2)	51	9	
N1/N2/N3	8 (11.8)	3	5	
Pathological stage				< 0.001
I/II	55 (80.9)	50	5	
III	13 (19.1)	4	9	
TTF-1 expression				0.001
Positive	33 (48.5)	32	1	
Negative	35 (51.5)	22	13	
CK-20 expression				0.189
Positive	19 (27.9)	16	3	
Negative	49 (72.1)	38	11	
CK 7 expression				1.000
Positive	66 (97.1)	52	14	
Negative	2 (2.9)	2	0	

*TTF-1* thyroid transcription factor-1, *CK* cytokeratin

### Survival analyses between patients with solitary-type and those with pneumonic-type based on CT findings

**Fig. 3:**
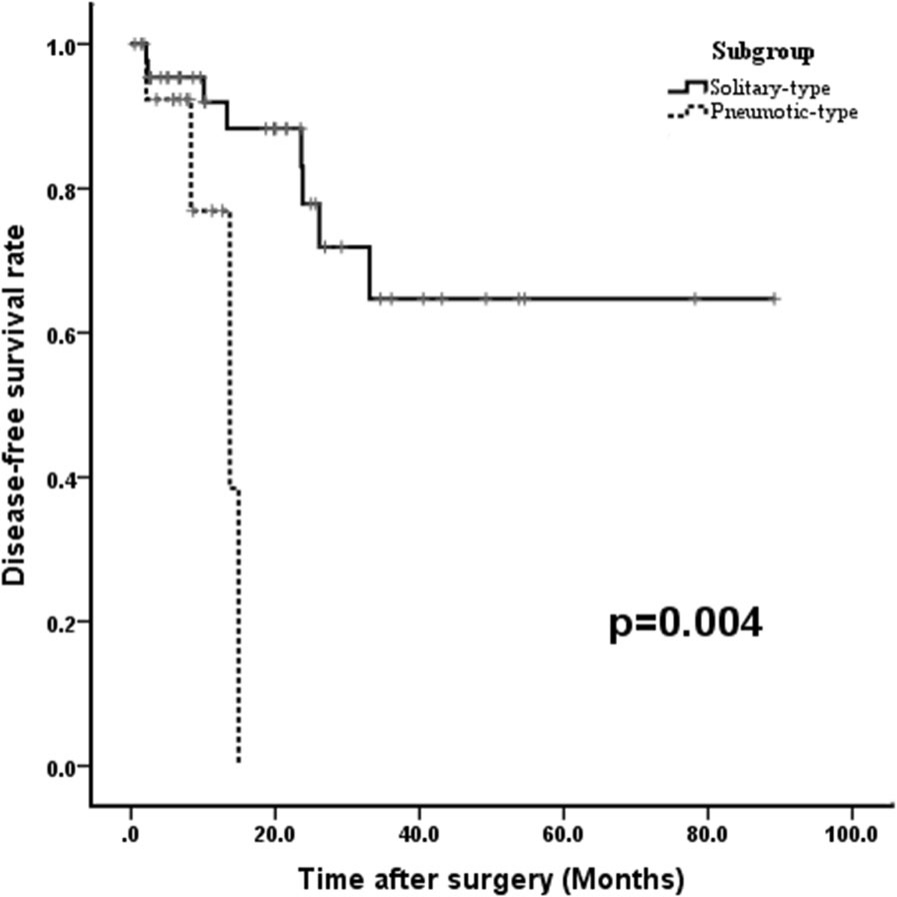
Disease-Free survival curves for patients with solitary-type (solid line) and pneumonic-type (dashed line) tumors

To investigate the effects of radiological subtype on the survival, we compared the survival rate between the patients with solitary-type (thereafter named solitary group) and those with pneumonic-type (thereafter named pneumonic-type group). As a result, the median follow-up time was 10.75 (range, 1.4–89.2) months. The 5-year DFS rate was 64.7 and 0% for solitary group and pneumonic-type group, respectively. Compared with the solitary group, the pneumonic-type group showed a significantly poorer DFS (*P* = 0.004) (Fig. 3). Furthermore, 9 patients developed cancer recurrence and/or metastases after resection in pneumonic-type group. The initial metastases were intrathoracic metastases, including lung metastasis, pleural dissemination, and mediastinal lymph node metastasis.

### Risk factors correlated with postoperative prognosis

According to univariate analysis with Cox proportional hazards model for DFS, T stage, pathological stage, TTF-1 expression, and pneumonic pattern on CT scan were significant factors correlated with DFS (*P* = 0.014, *P* = 0.013, *P* = 0.029, *P* = 0.011, respectively) (Table 3). Multivariate analysis showed that pneumonic-type on CT scan was an independent prognostic factor for DFS [hazard ratio (HR) = 6.764, 95% confidence interval (CI): 1.563–29.269, P = 0.011], indicating that patients in pneumonic-type group had a worse survival outcome and higher risk for recurrence compared with those in solitary group (Table 3).

**Table 3:** Univariate and Multivariate Analyses with Cox Proportional Hazards Model for Disease-free Survival

Prognostic factors	Univariate analysis			Multivariate analysis		
	HR	95% CI	*P* value	HR	95% CI	*P* value
Age (> 65 vs. ≤ 65)	3.180	0.955–10.589	0.059	…	…	…
Gender (Male vs. Female)	0.618	0.199–1.919	0.406	…	…	…
Smoking history (Yes vs. No)	1.177	0.354–3.912	0.790	…	…	…
T stage (T3~4 vs. T1~2)	5.552	1.411–21.842	0.014	…	…	…
N stage (N1~3 vs. N0)	2.636	0.286–24.260	0.392	…	…	…
Pathological stage (III vs.I + II)	5.467	1.428–20.930	0.013	…	…	…
TTF-1 (+vs.-)	0.239	0.066–0.861	0.029	…	…	…
CK20 (+vs.-)	0.984	0.265–3.661	0.981	…	…	…
CT feature, pneumonic type (Yes vs. No)	6.764	1.563–29.269	0.011	6.764	1.563–29.269	0.011

*HR* Hazard Ratio, *95%CI* 95%Confidence Interval, *TTF-1* thyroid transcription factor-1, *CK* cytokeratin

*P* value, from Wald Chi-Square Test in Cox Regression

## Discussion

The aim of this study was to distinguish the differences between clinicopathological characteristics and prognosis of subtypes of IMA according to CT findings. Based on the 2011 ATS/IASLC/ERS classification system for lung adenocarcinoma, several scholars have already demonstrated that system has a clear prognostic significance. IMAs have been considered to have worse prognosis than lung non-mucinous adenocarcinoma [16, 17]. Several previous cohort studies have indicated that pure IMA have better prognosis compared with mixed mucinous/nonmucinous IMA, and that mixed mucinous/ nonmucinous pattern is an independent risk factor for DFS [13, 14]. To date, previous studies have focused on the influence of the pathological classification on prognosis and genetic findings [3, 13], in which the differences between pure IMA subgroups based on CT findings and optimal management for IMA have not been precisely elucidated. Recent researches [10, 11] have indicated that thin-section CT images for IMAs could be helpful for predicting prognosis before surgical resection. Based on CT findings, Shimizu et al. [10] have divided lung IMA into three subtypes, including solid, bubbling, and pneumonic types, and have reported that the pneumonic-type was correlated with a significantly worse outcome compared with solid or bubbling type (*P* = 0.018). Nevertheless, their research cohort included only 29 IMA patients, suggesting that their observations were obviously limited by sample size. Furthermore, Watanabe et al. [11] have reported a series of 40 surgically resected patients with mucinous adenocarcinoma which were divided into two groups (pneumonic-type and solitary) based on the CT scans, with the similar outcome in survival analysis. Nonetheless, 14 out of 40 patients were mucinous AIS or MIA. Both subtypes showed 100% 5-year survival rate if they were fully resected. Therefore, we can conclude that it is not feasible to accurately assess the differences in clinicopathological features and prognosis of IMA subgroups based on CT scan.

In the current cohort, all patients were diagnosed with pure IMA. The IMAs were classified into two groups, involving pneumonic-type group (*n* = 14) and solitary group (*n* = 54), in which the majority of patients were in stage I/II. Importantly, it was revealed that within 11.8% of the patients showed lymph node metastasis, and 88.2% of the patients had nodal stage N0, while there was no case with extrathoracic metastasis. Further statistical analysis showed that pneumonic-type group had a significantly higher rate of lymph node metastasis than solitary group (*P* = 0.008). These data were consistent with several previous studies [12, 18–20]. Tsuta et al. [20] reported that the lymph node-positive rate was 6.8%, which was lower compared with other subtypes of lung adenocarcinoma, such as acinar, papillary, micropapillary, and solid predominant patterns. Because lymph node metastasis is associated with poorer prognosis, it can be used to explain the unfavorable outcome in patients with pneumonic-type adenocarcinoma.

In our cohort, it was revealed that IMA patients were almost older, female (60.3%), non-smoker (70.6%), as well as asymptomatic when were initially diagnosed (64.7%). However, no significant differences in demographic characteristics were observed between types of tumor on CT. These findings were consistent with the results reported by previous studies [11, 12, 19, 21], suggesting that IMA mainly occurs at a greater frequency in female and non-smoking patients. In the majority of cases, it may be difficult to distinguish pneumonia and pneumonic types of IMA based on initial CT findings. In the present study, we found that the excessive coughing with excessive white sputum was more common in patients with pneumonic-type compared with that with solitary-type. These data were consistent with a previous study as well [11]. This might be due to that the pneumonic-type was associated with higher stage and uncontrolled growth of tumor cells, which may cause excessive production of mucus and its discharge through the upper airways. The symptom of coughing with excessive white sputum may help early diagnosis of pneumonic-type adenocarcinoma, while the correlation between symptoms and pathology needs to be further studied.

According to the 8th edition of the TNM staging classification, tumor size is considered as the most important prognostic factor. Tumor being greater than 5 cm, that is defined as T3/4 stage, is taken as a high risk factor into account. In the current study, according to univariate analysis of 68 IMA patients, there was a significant worse DFS for T stage (T3~4 versus T1~2), TTF-1 (positive versus negative), pathological stage (III versus I + II) and pneumonic type (Yes versus No). Pneumonic-type showed larger tumor size and more lymph node metastases compared with solitary-type. Therefore, the TNM classification and pathological stage were significantly higher in pneumonic-type group compared with solitary group; besides, pneumonic types had a significantly poorer survival time for DFS. These data were consistent with other studies [10–12]. With reference to poor survival outcome, we found that pneumonic-type was an independent risk factor for poor survival in patients with IMA, while age, T stage, N stage, TTF-1, and pathological stage in our study were not independent predictors of DFS on the multivariate analysis. Recently, Lee et al. [12] reported a cohort of IMA patients and found that both tumor size (HR = 1.370, 95% CI: 1.141–1.645, *P* = 0.001) and SUV_max_ (HR = 1.338, 95% CI: 1.160–1.544, *P* < 0.001) were significant independent poor prognostic predictors for DFS, however, the consolidative pattern based on CT features was not related to DFS (*P* = 0.280). This could be due to the TNM stage of their involved IMA patients indicated based on the 7th edition of the TNM classification system and the rate of lymph node metastasis of consolidative type patients was 0%; additionally, different CT features and clinical relevance of covariates were compared with the present study.

TTF-1 expression is an important prognostic indicator of lung adenocarcinoma. Winslow et al. [22] found that increased expression of TTF-1 suggests a better prognosis, while decreased expression increases tumor colonization and metastatic ability. Previous studies [23, 24] have also demonstrated that TTF-1 positive expression is an important prognostic factor for lung adenocarcinoma. According to previous researches, the majority of the IMAs expressed CK7 (~ 88%) and CK20 (~ 54%) [25–27]. However, the expression of TTF-1 rate is different in the IMA, and its expression rate in IMA is often lower than that in non-mucinous adenocarcinoma [28, 29]. Our data indicated that the DFS was significantly different between IMA patients with positive and negative expressions of TTF-1 (*P* = 0.029). The DFS of patients with negative expression of TTF-1 was worse than those with positive expression. In addition, in this group of cases, the expression of TTF-1 in pneumonia-type IMA was mostly negative, with only one positive case, which may be related to the advanced stage of the pneumonia-type IMA. Zhang et al. [30] reported that negative expression of TTF-1 tends to be more frequent in higher stage tumors, which is consistent with our findings.

The present study has several limitations. Firstly, it was a retrospective study with relatively small sample size. Secondly, only DFS was considered, while overall survival was not calculated, which should be addressed by further studies. Moreover, our survival analysis did not examine the status of gene mutation in all the patients in the present cohort, suggesting that further study of molecular data should be conducted.

## Conclusions

Compared to Pneumonic-type IMA, the Solitary-type IMA is associated with a lower stage and better prognosis. Besides, pneumonic-type is a significant independent risk factor for shorter DFS in patients with IMA.

## Data Availability

The datasets analysed during the current study are available from the corresponding author on reasonable request.

## Abbreviations

CT: Computed tomography
IASLC/ATS/ERS: International Association for the Study of Lung Cancer/American Thoracic Society/European Respiratory Society
IMA: Invasive mucinous adenocarcinoma
NSCLC: Non-small cell lung cancer
